# Bulky hydrophobic side chains in the β1-sandwich of microsomal triglyceride transfer protein are critical for the transfer of both triglycerides and phospholipids

**DOI:** 10.1016/j.jbc.2024.105726

**Published:** 2024-02-05

**Authors:** Narasimha Anaganti, Swati Valmiki, Rosario Recacha, Shahidul Islam, Steven Farber, Lloyd Ruddock, M. Mahmood Hussain

**Affiliations:** 1 Department of Foundations of Medicine, NYU Grossman Long Island School of Medicine, Mineola, New York, USA; 2 Faculty of Biochemistry and Molecular Medicine, University of Oulu, Oulu, Finland; 3 Department of Biology, Johns Hopkins University, Baltimore, Maryland, USA

**Keywords:** apolipoprotein B, lipoprotein, lipids, lipid transfer, mutagenesis

## Abstract

Hyperlipidemia predisposes individuals to cardiometabolic diseases, the most common cause of global mortality. Microsomal triglyceride transfer protein (MTP) transfers multiple lipids and is essential for the assembly of apolipoprotein B-containing lipoproteins. MTP inhibition lowers plasma lipids but causes lipid retention in the liver and intestine. Previous studies suggested two lipid transfer domains in MTP and that specific inhibition of triglyceride (TG) and not phospholipid (PL) transfer can lower plasma lipids without significant tissue lipid accumulation. However, how MTP transfers different lipids and the domains involved in these activities are unknown. Here, we tested a hypothesis that two different β-sandwich domains in MTP transfer TG and PL. Mutagenesis of charged amino acids in β2-sandwich had no effect on PL transfer activity indicating that they are not critical. In contrast, amino acids with bulky hydrophobic side chains in β1-sandwich were critical for both TG and PL transfer activities. Substitutions of these residues with smaller hydrophobic side chains or positive charges reduced, whereas negatively charged side chains severely attenuated MTP lipid transfer activities. These studies point to a common lipid transfer domain for TG and PL in MTP that is enriched with bulky hydrophobic amino acids. Furthermore, we observed a strong correlation in different MTP mutants with respect to loss of both the lipid transfer activities, again implicating a common binding site for TG and PL in MTP. We propose that targeting of areas other than the identified common lipid transfer domain might reduce plasma lipids without causing cellular lipid retention.

Microsomal triglyceride transfer protein (MTP) is a heterodimeric protein consisting of a 98 kDa larger MTP α-subunit and a 55 kDa smaller protein disulfide isomerase (PDI) β-subunit (1, 2, 3). MTP subunit confers lipid transfer function, whereas the PDI subunit is hypothesized to keep the MTP subunit in solution and retain within the endoplasmic reticulum (4, 5). In the endoplasmic reticulum, MTP is hypothesized to transfer various lipids to nascent apolipoprotein B (apoB) to initiate the formation of intestinal chylomicrons and liver very low–density lipoproteins (3, 4, 6, 7, 8). The absence of functional MTP results in the degradation of nonlipidated apoB leading to abetalipoproteinemia that is characterized by the absence of plasma lipoproteins (9, 10, 11, 12). More than 75 mutations have been identified in the *MTTP* gene of abetalipoproteinemia subjects (13). Analyses of several missense mutations causing amino acid substitutions in abetalipoproteinemia revealed that these mutants lack lipid transfer activities and do not support apoB-lipoprotein secretion (14, 15). Thus, it is now well established that the lipid transfer activity of MTP is crucial for apoB-lipoprotein biosynthesis.

MTP has been shown to transfer several lipids including triglycerides (TGs) and phospholipids (PLs) (7, 8, 16, 17). Evolutionary studies suggest that MTP evolved as a PL transfer protein in invertebrates and acquired TG transfer activity at the base of vertebrate evolution (18). The MTP in *Drosophila* (invertebrates) possesses only PL transfer activity and lacks TG transfer activity (19) and this PL transfer activity has been shown to be sufficient to support apoB secretion in cells and mice (20, 21). In contrast, vertebrate MTP transfers both PL and TG. It is unclear whether the PL transfer domain acquired the ability to recognize TG or a different domain evolved for TG transfer. A recent study found that a mutation in zebrafish MTP (G863V) resulted in a significant loss of TG transfer activity but had little effect on PL transfer activity. The orthologous mutation in human MTP (hMTP; G865V) resulted in similar loss of TG but not PL transfer activity (22). The crystal structure of hMTP suggests the presence of two β-sandwiches; β1-sandwich comprised of A- and C-sheets within the C-terminal β-barrel surrounds a hydrophobic cavity of sufficient size to encapsulate lipids and β2-sandwich comprised of A-sheet and N-terminal β-barrel whose edge is lined with positively charged amino acids (2). Therefore, we hypothesized that the hydrophobic cavity in the β1-sandwich may interact with hydrophobic neutral lipids, such as TG, whereas the positively charged amino acids in the β2-sandwich may interact with the negatively charged phosphate groups of PLs. Kinetic studies by Atzel *et al.* (23) also indicate for the presence of two lipid-binding sites in MTP. Thus, biochemical, structural, and kinetic evidence indicate the possible existence of two different sites in MTP for the transfer of PL and TG. However, systematic studies to identify the different domains and amino acids crucial for specific lipid transfer activities have not been performed, and there is paucity of information about the location of different lipid transfer domains in MTP.

Worldwide, hyperlipidemias, obesity, and associated metabolic dysregulations are common major causes of significant global morbidity and mortality (24, 25, 26, 27, 28, 29). Dirlotapide, implitapide, and lomitapide (30), which inhibit all lipid transfer activities of MTP, have been used to reduce obesity and hyperlipidemia in humans and dogs, but they cause lipid accumulation in the liver and intestine (31, 32). As summarized previously, there is preponderance of evidence for the existence of different lipid transfer domains in MTP. Therefore, identifying the relevant binding sites for TG and PL in MTP may facilitate in the development of specific inhibitors targeting only TG transfer while retaining PL transfer activity. Here, we tested a hypothesis that β1-sandwich and β2-sandwich may be involved in the transfer of TG and PL, respectively.

To identify the amino acids and domains important for the transfer of these lipids, we used site-directed mutagenesis to generate missense mutations (substitutions) in different domains of MTP and assessed their effects on various MTP functions; TG and PL transfer activities, PDI interaction, and apoB secretion. In this study, we report 38 mutations in the larger MTP subunit and show that substitution of charged residues in the predicted PL transfer domain (β2-sandwich) does not affect MTP function. However, point mutations, especially substitutions of charged amino acids, in the hydrophobic cavity of β-sandwich-1 severely affect both TG and PL transfer activities indicating for the existence of a common site for the transfer of these lipids.

## Results

### General strategy

**Figure 1:**
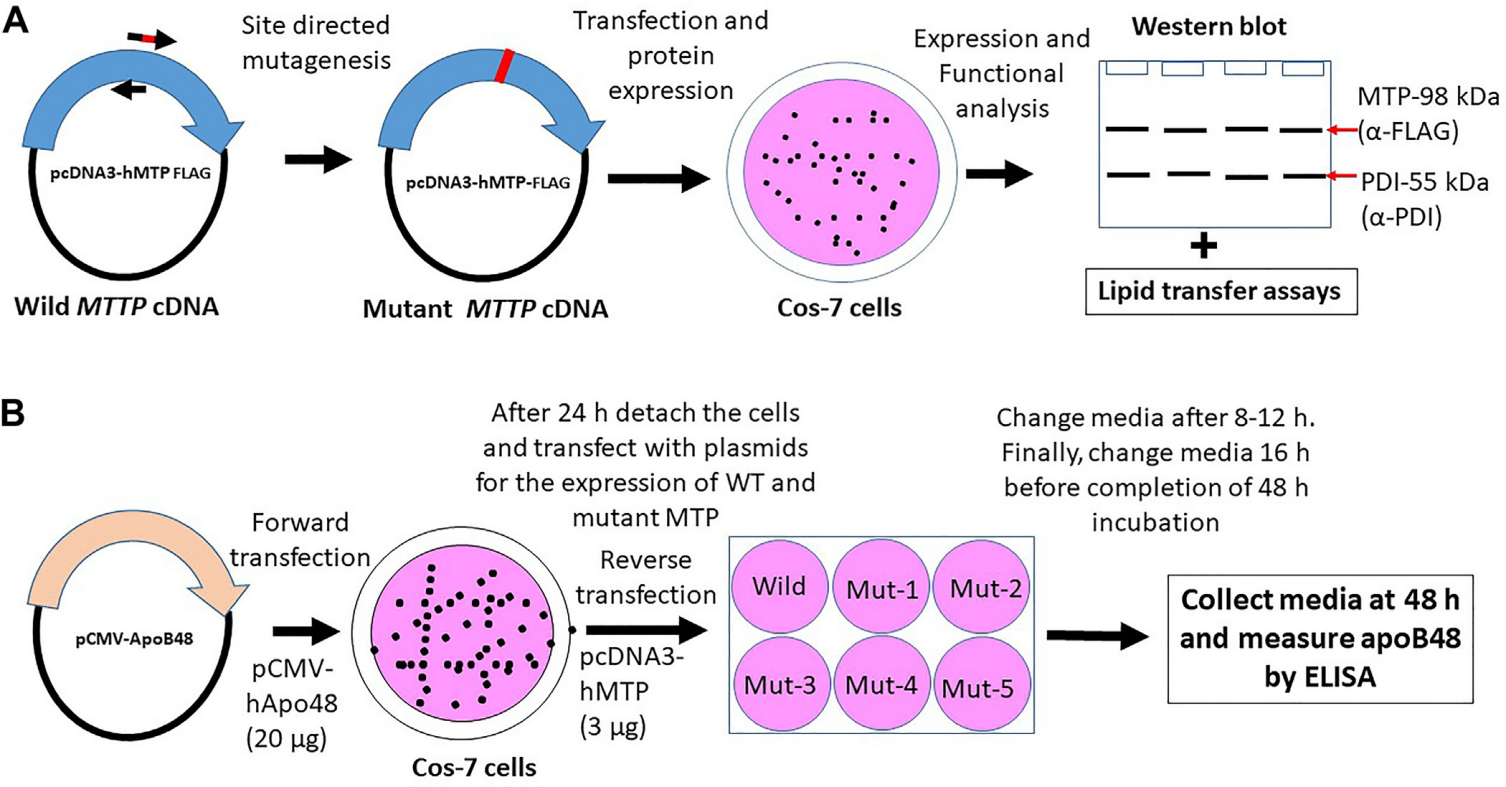
**Schematic diagrams depicting experimental strategy.***A*, mutagenesis and analyses of different MTP mutants. Site-directed mutagenesis was used to create specific missense mutations in human MTP-FLAG plasmid. Specific mutations were confirmed by sequencing. These plasmids were transfected into Cos-7 cells. Protein expression in Cos-7 cells was confirmed by Western blotting. Furthermore, WT and mutant MTP proteins along with PDI were copurified using anti-FLAG antibody beads to study PDI binding and to measure different lipid transfer activities. *B*, protocol to assess ability of different MTP mutants to support apoB48 secretion. Cos-7 cells were first transfected with plasmids expressing human apoB48. These cells were then trypsinized and distributed into different 6-well plates containing MTP plasmid and EndoFectin complexes for reverse transfection. After 48 h, media were collected to measure apoB by ELISA. Cells were collected to measure total proteins. ApoB secretion was normalized to cellular protein. MTP, microsomal triglyceride transfer protein; Mut, mutant; PDI, protein disulfide isomerase.

The aim of this study was to identify important domains and amino acid residues in hMTP that participate in TG and PL transfer. We first identified different functional domains based on the published crystal structure of hMTP (2). Second, individual amino acids within these domains were mutated to change charge or hydrophobicity of specific amino acids in plasmids expressing hMTP with a C-terminal FLAG tag (14, 15, 21). Third, plasmids for the expression of C-terminal FLAG-tagged WT and mutant hMTP proteins were introduced in monkey kidney Cos-7 cells, which do not express MTP and apoB. Fourth, expression of different MTP mutants was compared in cell lysates using anti-FLAG antibodies (Fig. 1*A*). We used anti-FLAG antibodies as the FLAG epitope is likely to be unperturbed by specific mutations in the MTP protein based on previous studies (14, 15). Fifth, we purified WT and mutant hMTP-FLAG proteins using anti-FLAG immune-affinity beads (15, 33). The purified proteins were again subjected to Western blot analyses, and protein densities were quantified to determine the association of PDI with the MTP subunit (Fig. 1*A*). Sixth, the purified proteins were used to measure TG and PL transfer activities of MTP. Seventh, to address the ability of WT and mutant hMTP proteins to support apoB48 secretion, Cos-7 cells were first transfected with apoB48 expression plasmid. Cells were then distributed equally in 6-well plates that contained plasmids for the expression of WT or mutant hMTP proteins and EndoFectin complexes for reverse transfection (Fig. 1*B*). Media were used to measure secreted apoB48 using human-specific monoclonal antibody, 1D1, as a capture antibody (34, 35). ApoB levels were normalized with cell protein, and the ability of different mutant proteins to support apoB48 secretion was evaluated.

### Different lipid-binding sites in MTP structure

**Figure 2:**
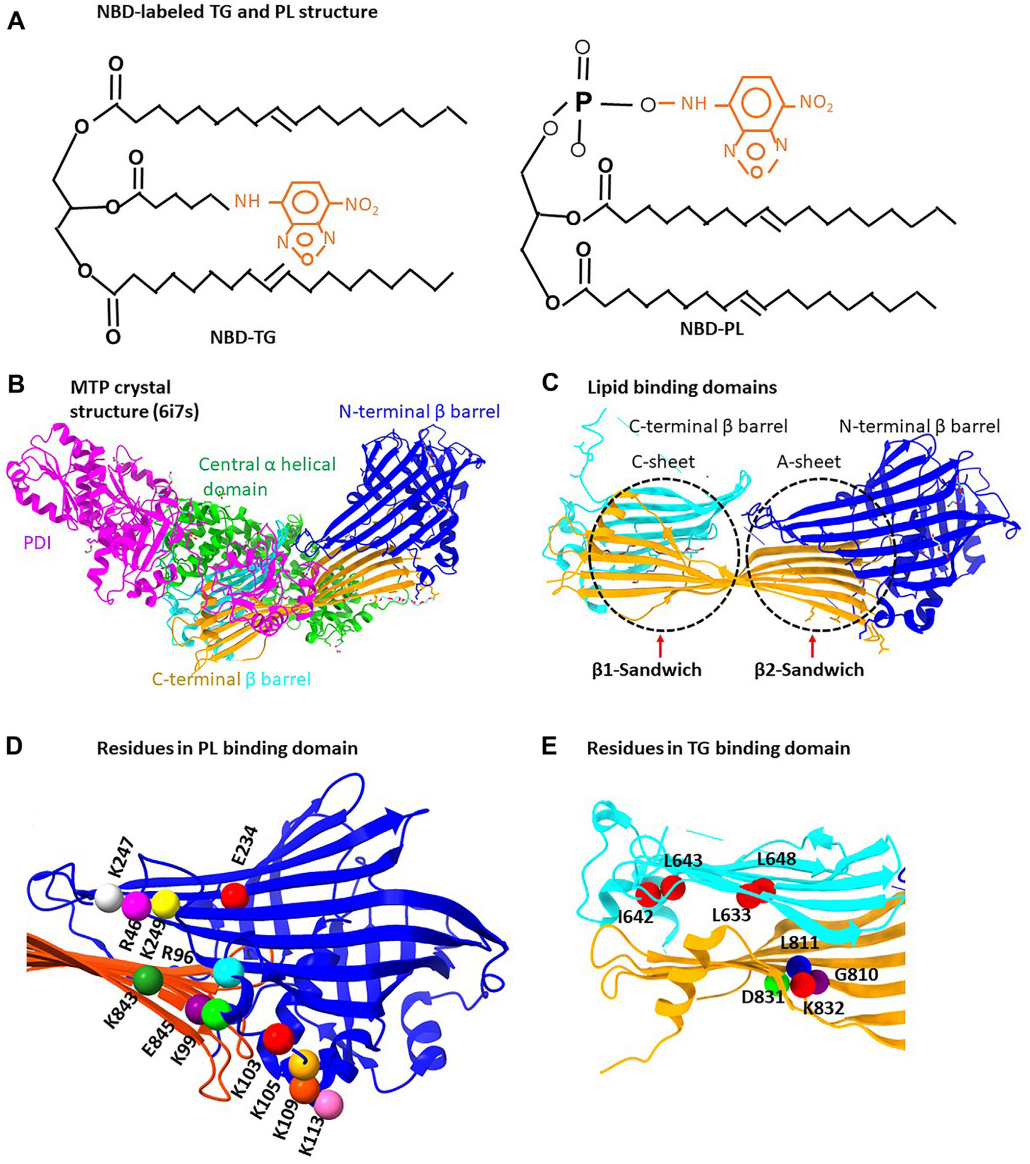
**Identification of important domains and residues for MTP function based on crystal structure.***A*, schematic representation of NBD-labeled lipids used as surrogate for TG (1,3-Di(*cis*-9-octadecenoyl)-2-((6-(7-nitrobenz-2-oxa-1,3-diazol-4-yl)amino)hexanoyl)-glycerol) and PL [1,2-dioleoyl-*sn*-glycero-3-phospho-ethanolamine-*N*-(7-nitro-2-1,3-benzoxadiazol-4-yl) (ammonium salt)] transfer. *B*, the MTP crystal structure (6i7s) was redrawn using Chimera software to illustrate different domains of MTP. *Pink color* denotes the PDI subunit. The MTP subunit consists of N-terminal β-barrel (*blue*), central α-helical domain (*green*), and C-terminal β-barrel consisting of A-sheet (*yellow*) and C-sheet (*cyan*). *C*, the lipid-binding domains in MTP are hypothesized to consist of two sandwiches; the β1-sandwich is between A-sheet and C-sheet in the C-terminal β-barrel, and β2-sandwich is between A-sheet (*yellow*) and N-terminal β barrel (*blue*). *D*, PL-binding domain. To test the hypothesis that negatively charged phosphate groups in PLs may interact with positively charged side chains, several charged residues (lysine, K; glutamic acid, E; and arginine, R) were identified. *E*, the predicted TG-binding domain is hypothesized to be between A-sheet (*yellow*) and C-sheet (*cyan*) and is rich in hydrophobic leucine residues. Some of the residues interrogated in this region have been identified. There are two charged residues, D831 and K832, in this domain. These were also mutated to determine whether substitutions of these residues affect the lipid-transfer activities of MTP. MTP, microsomal triglyceride transfer protein; NBD, nitrobenzoxadiazole; PL, phospholipid; TG, triglyceride.

PL and TG differ in two fundamental aspects. First, TG has three hydrophobic acyl groups attached, whereas PL has only two, hence the volume of PL is smaller. Second, TG has a neutral head group, whereas PL has negatively charged head groups. These differences make TG more hydrophobic than PL and suggest that there may be two distinct lipid-binding sites. In this study as before (14, 15, 33, 36, 37, 38), we used nitrobenzoxadiazole (NBD)-TG and NBD-PL as surrogates for TG and PL transfer, respectively (Fig. 2*A*). The structure of hMTP has three distinct structural domains: N-terminal β-barrel, central α-helical domain, and C-terminal β-barrel (Fig. 2*B*). MTP has two separate β-sandwiches, and here, we tested the hypothesis that one may bind TG and the other PL (Fig. 2*C*). The β1-sandwich is formed from the C-sheet (amino acids 604–717) and the A-sheet (amino acids 722–886) in the C-terminal β-barrel (Fig. 2*C*). In the published structure, this encloses a hydrophobic region occupied by a molecule of polyethylene glycol. The volume of this cavity is approximately 2100 Å^3^, which is sufficient to envelope a TG (the volume of triolein is approximately 1600 Å^3^). The β2-sandwich is formed by the A-sheet of the C-terminal β-barrel and the N-terminal β-barrel (amino acids 18–297) (Fig. 2*C*). In the published structure, there is no cavity between these sheets, but it was speculated that a conformational change could result in the formation of a cavity of slightly smaller size to that found in β1-sandwich. If TG and PL bind in different β-sandwiches, we hypothesized that binding of the charged headgroups in PL may require electrostatic interactions with positively charged residues in MTP (Fig. 2*D*). β1-Sandwich is very hydrophobic, and there is only a single positively charged residue, K832, located nearby, which could interact with the PL headgroup (Fig. 2*E*). In contrast, there are a large number of positively charged groups lining the edge of β2-sandwich (Fig. 2*D*). We therefore hypothesized that TG may be bound by β1-sandwich and PL by β2-sandwich. Furthermore, we hypothesized that positively charged groups in the β2-sandwich are critical for PL transfer activity. To test these hypotheses, we mutated specific residues, and purified mutant proteins were used to evaluate their role in MTP lipid transfer function. Finally, the effects of different mutations were also analyzed using Sorting Intolerant From Tolerant (SIFT), PolyPhen, and Panther online bioinformatic tools that predict the effects of different missense mutations on protein function and were compared with our experimental data.

### Positively charged amino acids in β2-sandwich are not critical for PL transfer

**Figure 3:**
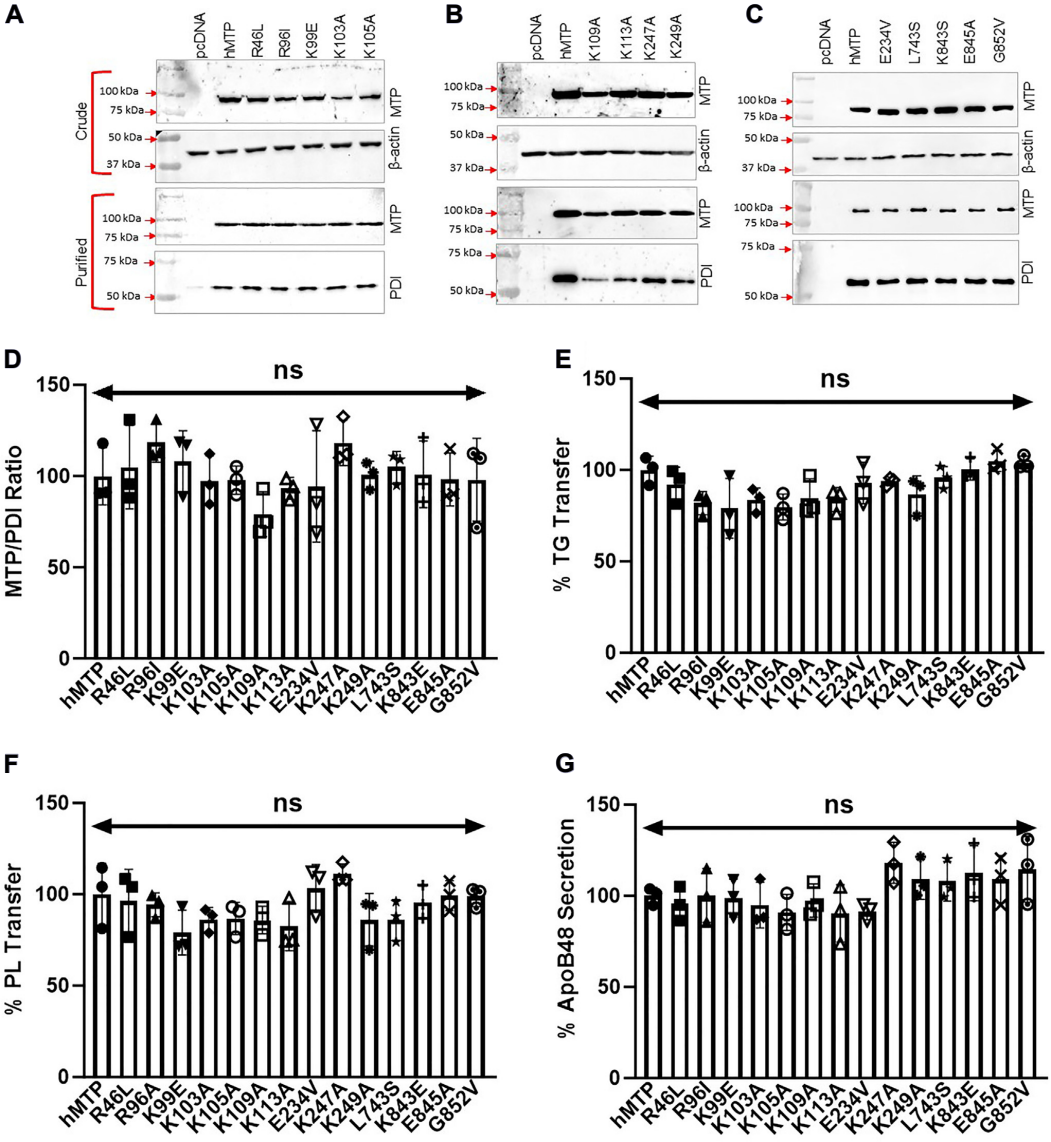
**Amino acid residues with charged side chains in the predicted PL-binding domain of MTP are not critical for PL transfer.** The amino acid residues at R46, R96, K99, K103, K105, K109, K113, E234, K247, K249, L743, K843, E845, and G852 were mutated as shown. These mutants were independently transfected in triplicate in Cos-7 cells. After 48 h, cells were lysed and MTP protein was purified as explained in the Experimental procedures section. *A*–*C*, Western blots of MTP and β-actin in crude protein fractions (*top*, 30 μg). MTP and PDI detection in purified fractions (*bottom*, 30 μl). The proteins were separated on 8% SDS-PAGE gels and blotted onto nitrocellulose membranes and probed with mouse anti-FLAG antibodies. The loading control β-actin (*bottom*) was probed with rabbit anti-β-actin antibody. Data for one set are shown. *D*, Western blots of MTP and PDI subunits in purified MTP (*A*–*C*, *bottom*) were quantified by ImageJ, and ratios were calculated. Ratios for different mutants were plotted normalized to WT hMTP. *E*, purified MTP proteins (90 μl) were used to measure TG transfer activity in technical duplicates. Data from the 60 min time point measured in duplicates are plotted as % of WT hMTP control. *F*, PL transfer activities of hMTP variants measured in technical duplicates at 240 min plotted as a % of the WT hMTP control. *G*, Cos-7 cells were first transfected with plasmid for the expression of human apoB48. After 24 h, cells were trypsinized and reverse transfected with WT or different mutant hMTP-expressing plasmids (n = 3). ApoB48 concentrations in the media were measured in duplicate. Values in the WT hMTP-transfected cells were normalized to 100%, and effects of different mutants were compared with this control. The data are representative of three independent experiments. The statistical significance was calculated by one-way ANOVA (multiple comparisons). The error bars represent SD. The symbols ∗, ∗∗, ∗∗∗, and ∗∗∗∗ represent significance at *p* < 0.05, *p* < 0.01, *p* < 0.001, and *p* < 0.0001, respectively. hMTP, human MTP; MTP, microsomal triglyceride transfer protein; PL, phospholipid; TG, triglyceride.

We first interrogated few negatively charged and uncharged residues in this region. The negatively charged amino acids at E234 and E845 were substituted with hydrophobic residues valine and glycine, respectively. The L743, a hydrophobic residue, was replaced with serine. At 852 position, the small hydrophobic residue glycine was changed to larger hydrophobic residue valine. All these four mutations had no effect on MTP–PDI interactions, the MTP lipid transfer activities, and apoB48 secretion (Fig. 3, *A*–*G*).

**Figure 4:**
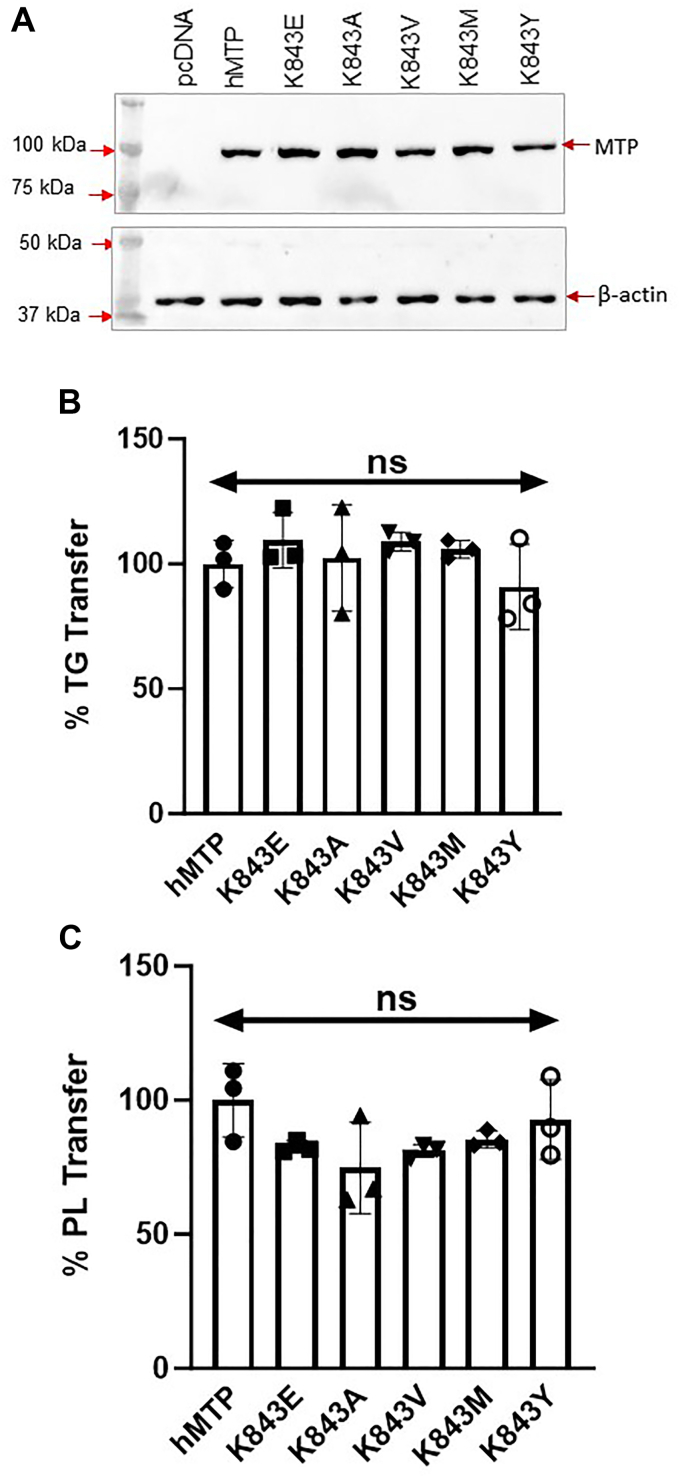
**Effect of different amino acid substitutions at K843 on MTP function.***A*, the protein expression of various K843 variants of MTP large subunit in Cos-7 cell crude extracts (*top*). β-actin was used for loading control (*lower*). *B* and *C*, TG and PL transfer activities of K843 variants at 60 and 240 min, respectively, were measured in the crude extracts. MTP, microsomal triglyceride transfer protein; PL, phospholipid; TG, triglyceride.

Second, we tested the hypothesis that positive charges in the β2-sandwich may interact with negatively charged phosphate groups in PL to facilitate lipid binding and transfer. There is evidence that lysine and arginine residues in proteins interact with PL (39, 40). Substitution of R46, R96, K99, K103, K105, K109, K113, K247, K249, and K843, with different amino acids to remove positive charges had no effect on protein expression (Fig. 3, *A*–*C*) and their binding to PDI (Fig. 3, *A*–*D*). Next, we looked at TG and PL transfer activities. These mutations had no effect on TG and PL transfer activities (Fig. 3, *E* and *F*) indicating that these residues may not be critical for lipid transfer. Furthermore, these mutations had no effect on apoB48 secretion (Fig. 3*G*). To confirm that this was not because of the selected substitutions, we studied the effect of several other less conservative substitutions at K843. Substitution of K843 with glutamic acid, alanine, valine, methionine, or tyrosine had no effect on TG or PL transfer activities (Fig. 4, *A*–*C*). These studies indicated that these positively charged amino acids in β2-sandwich are not critical for PL transfer.

### Charged residues in β1-sandwich are also not critical for lipid transfer activities

**Figure 5:**
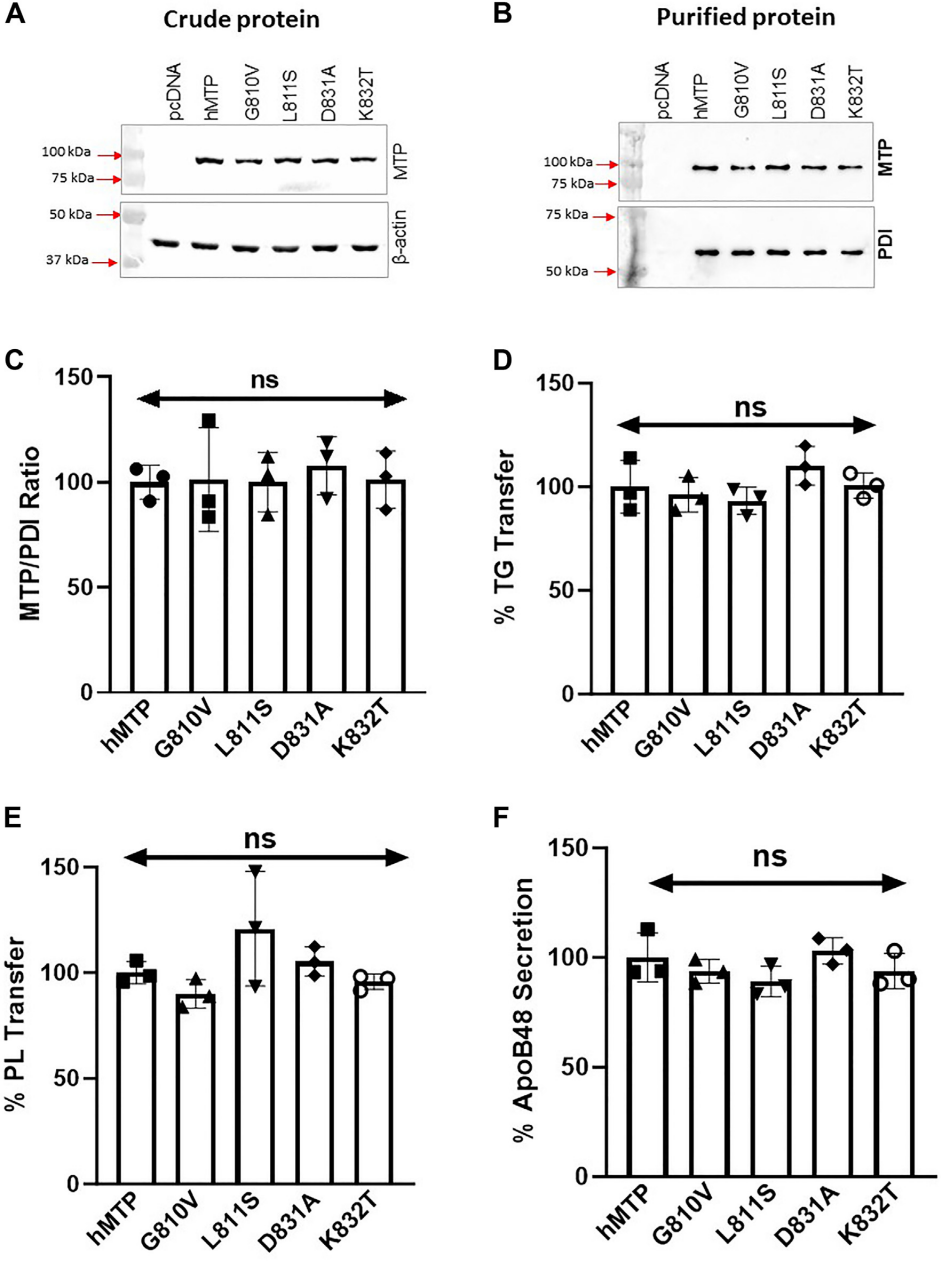
**Charged residues in β1-sandwich are not critical for MTP activity.***A*, mutations G810V, L811S, D831A, and K832T were made in a plasmid expressing human MTP. They were expressed in triplicate in Cos-7 cells. Western blots of MTP and β-actin in crude protein fractions in one set of cells are shown. *B*, MTP and PDI subunits were detected in purified MTP variants. *C*, MTP–PDI ratios in purified MTP proteins were calculated from Western blots in *B* after ImageJ scanning. *D*, TG transfer activities in purified proteins were measured at 1 h in duplicate. *E*, PL transfer activities in purified proteins were measured at 4 h in duplicate. *F*, ApoB48 concentrations measured in duplicates in the media of Cos-7 cells transfected first with plasmids (n = 3) for the expression of human apoB48 followed by transfections with different indicated mutant MTP variants as described in the Experimental procedures section. All experiments were repeated at least three times. Individual values were compared with WT MTP by one-way ANOVA. The error bars represent SD. ∗*p* < 0.05, ∗∗*p* < 0.01, ∗∗∗*p* < 0.001, and ∗∗∗∗*p* < 0.0001. MTP, microsomal triglyceride transfer protein; PDI, protein disulfide isomerase; PL, phospholipid; TG, triglyceride.

There are two charged residues, D831 and K832, in the A-sheet that is part of the β1-sandwich (Fig. 2*E*). We mutated these residues to neutral residues as D831A and K832T. These mutations had no effect on protein expression, PDI binding, TG transfer, PL transfer, and apoB48 secretion (Fig. 5, *A*–*F*) indicating that mutations at these charged residues in the A-sheet are fairly well tolerated, and these residues are probably not critical for lipid transfer.

### Hydrophobic environment outlined by A- and C-sheets in β1-sandwich is critical for both lipid transfer activities

The importance of the A- and C-sheets in the TG transfer activity has been demonstrated by mutagenesis studies (2, 14, 15, 41). Here, we tested tolerability of charges and hydrophobic chain lengths for protein expression, TG and PL transfer activities, PDI binding, and apoB48 secretion. First, we concentrated on G810 and L811 in the A-sheet. G810V and L811S mutations increased and decreased hydrophobicity, respectively. These mutations had no effect on protein expression, PDI binding, TG transfer, PL transfer, and apoB48 secretion (Fig. 5, *A*–*F*). These studies indicated that G810 and L811 residues are not critical for MTP function.

**Figure 6:**
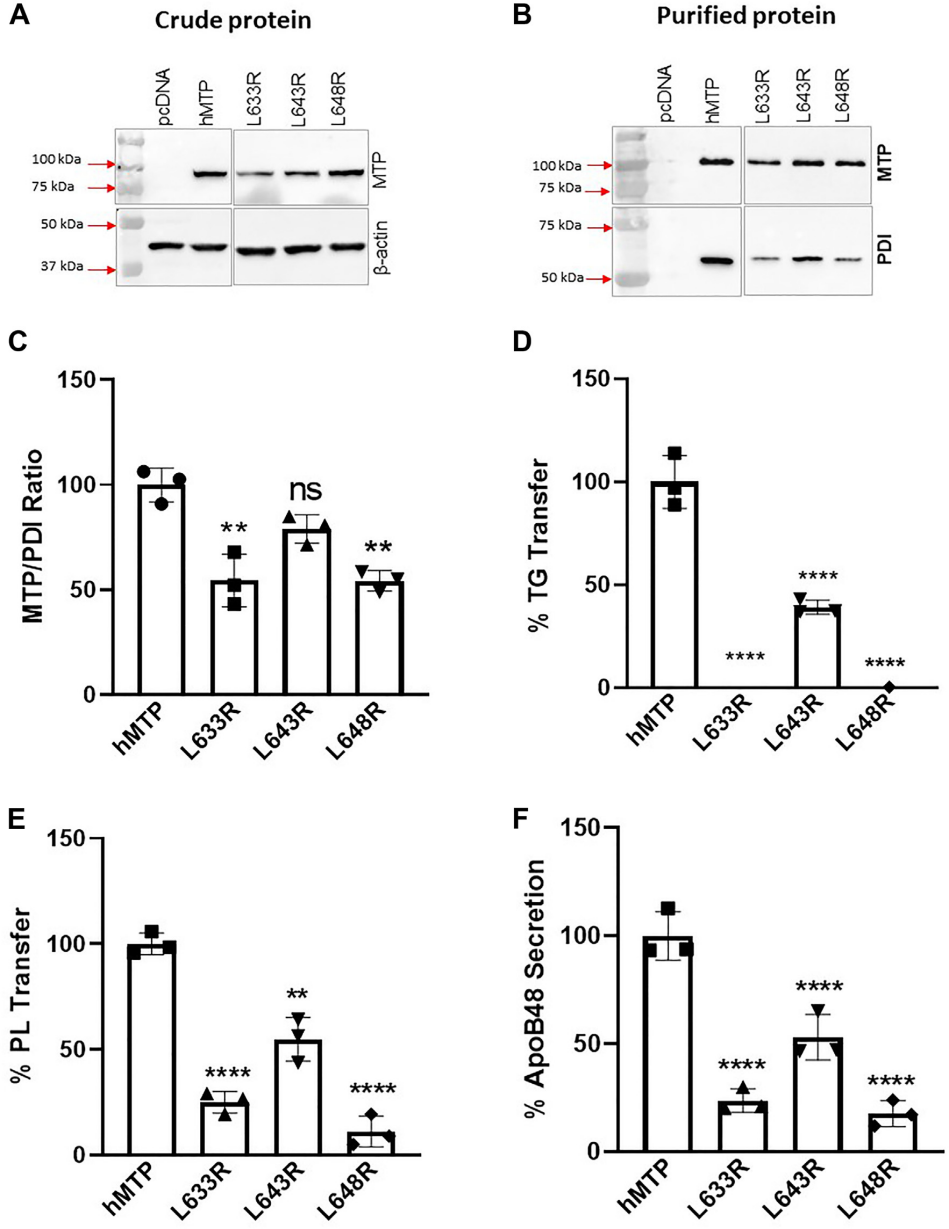
**Hydrophobic residues in the C-sheet are important for lipid transfer.***A*, mutations L633R, L643R, and L648R were made in a plasmid expressing human MTP. They were expressed in triplicate in Cos-7 cells. Western blots of MTP and β-actin in crude protein fractions in one set of cells are shown. *B*, MTP and PDI subunits were detected in purified MTP variants. *C*, MTP–PDI ratios in purified proteins were calculated from Western blots in *B*. *D*, TG transfer activities in purified proteins were measured at 1 h in duplicates. *E*, PL transfer activities in purified proteins were measured at 4 h in duplicate. *F*, ApoB48 levels in the media of Cos-7 cells transfected first with plasmids (n = 3) for the expression of human apoB48 followed by transfections with different indicated mutant MTPs as described in the Experimental procedures section. All experiments were repeated at least three times. Individual values were compared with WT MTP by one-way ANOVA. The error bars represent SD. ∗*p* < 0.05, ∗∗*p* < 0.01, ∗∗∗*p* < 0.001, and ∗∗∗∗*p* < 0.0001. MTP, microsomal triglyceride transfer protein; PDI, protein disulfide isomerase; PL, phospholipid; TG, triglyceride.

Next, we mutated hydrophobic leucine residues in the C-sheet (Fig. 2*E*). Mutagenesis of leucine residues at 633, 643, and 648 to arginine did not affect protein expression (Fig. 6*A*). L633R and L648R bound less to PDI (Fig. 6, *B* and *C*). L633R, L643R, and L648R mutations significantly reduced TG and PL transfer activities as well as apoB48 secretion (Fig. 6, *D*–*F*). These results suggested that these hydrophobic residues are critical for both lipid transfer activities as well as apoB48 secretion, and charged residues are not tolerated at these positions.

**Figure 7:**
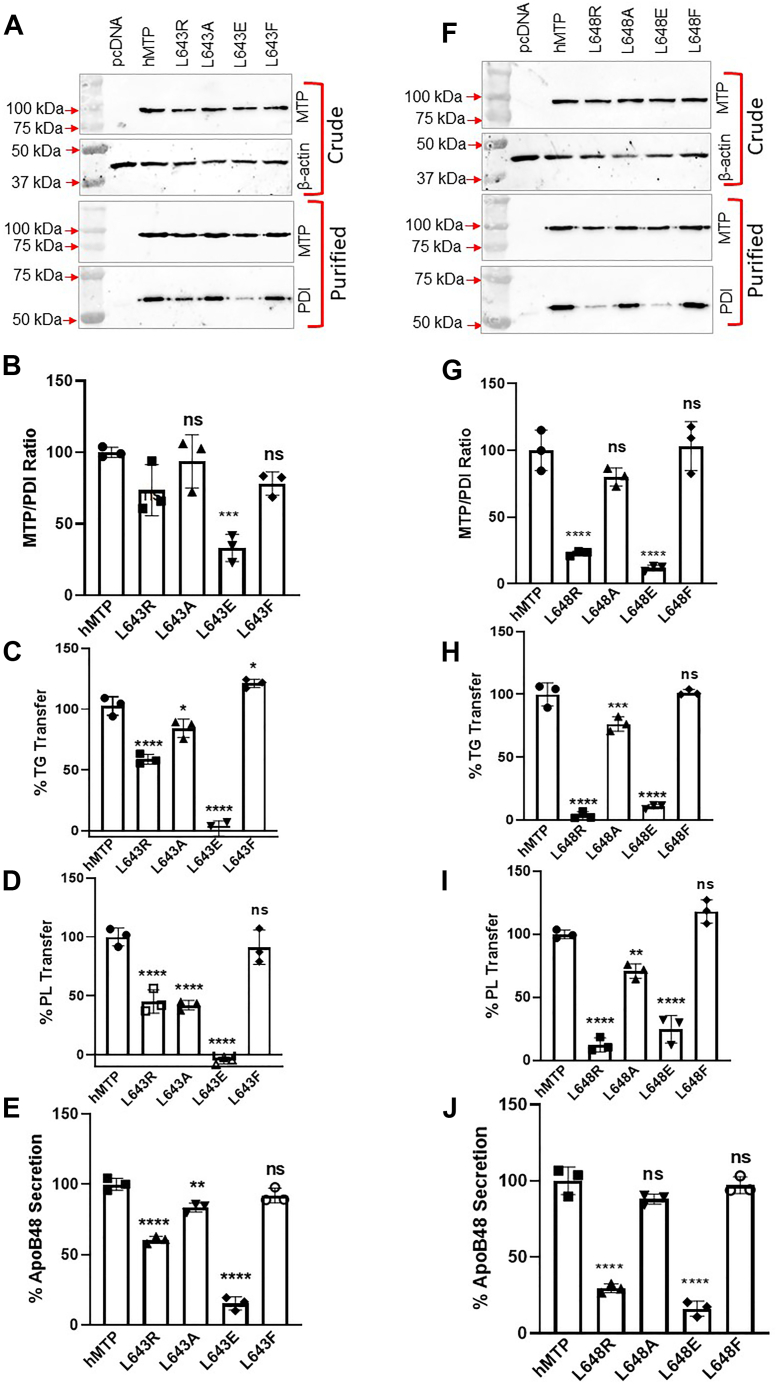
**Large hydrophobic side chains at amino acids 643 and 648 are important for MTP function.***A*–*E*, L643 was substituted with different indicated amino acid residues for investigation. These mutants were introduced in triplicate in Cos-7 cells for different analyses. *A*, Western blots of MTP and β-actin in crude protein fractions (*top*) and MTP and PDI in purified fractions (*bottom*) in one set of samples. *B*, the MTP–PDI stoichiometry was determined after scanning their levels in purified proteins. *C*, the TG transfer activity in different variants at 60 min. *D*, PL transfer activity of different mutants at 240 min. *E*, ApoB48 concentrations in the media of Cos-7 cells transfected with ApoB48 and MTP variants. *F*–*J*, different L648 mutants were studied to assess the importance of this residue in MTP function. *F*, L648 was mutated to different amino acids as indicated, and expression of different mutants (n = 3) was studied in crude cell extracts as well as after their purification. *G*, the MTP–PDI ratio was calculated from Western blots of purified proteins and normalized to this ratio in WT MTP. (*H*) TG and (*I*) PL transfer activities in purified MTP proteins were measured in duplicate. *J*, the ability of different mutants to support apoB48 secretion was assessed in Cos-7 cells in triplicates as described in the Experimental procedures section. The data presented here are representative of three independent experiments. The statistical significance of kinetics (*line graphs*) was calculated using one-way ANOVA (multiple comparison). The error bars represent SD. ∗*p* < 0.05, ∗∗*p* < 0.01, ∗∗∗*p* < 0.001, and ∗∗∗∗*p* < 0.0001. In few cases, error bars are not visible as values were below zero. MTP, microsomal triglyceride transfer protein; PDI, protein disulfide isomerase; PL, phospholipid; TG, triglyceride.

To address whether leucine residues or hydrophobic environments are critical for MTP function, we generated several mutants where L643 and L648 residues were substituted with arginine, alanine, glutamic acid, or phenylalanine to alter hydrophobic environment. These substitutions at L643 did not affect protein expression (Fig. 7*A*, *top*). Substitution of L643 with arginine, alanine, or phenylalanine had no effect on PDI binding, but substitution with glutamic acid reduced PDI binding (Fig. 7, *A* and *B*). Exchange of L643 with phenylalanine increased TG transfer activity (∼20%), whereas alanine slightly reduced TG transfer activity (∼15%) indicating that bulky hydrophobic groups at this site are optimal for TG transfer (Fig. 7*C*). L643R swap reduced TG transfer by ∼45%, whereas L643E completely abolished TG transfer activity, indicating a positive charge can be tolerated to some extent but a negative charge is deleterious. The PL transfer activity was unaffected by L643F (Fig. 7*D*). However, substitutions of alanine and arginine reduced PL transfer activity by greater than 50% (Fig. 7*D*), whereas L643E was unable to transfer PL (Fig. 7*D*). These studies indicated that bulky hydrophobic group of leucine at position 643 is critical for both TG and PL transfer. Decreases in hydrophobicity and changes to positive charges are somewhat tolerated, but negative charges are not. Testing for apoB48 secretion revealed that substitution of leucine with phenylalanine had no effect on apoB48 secretion (Fig. 7*E*). Alanine substitution slightly reduced (∼20%) apoB48 secretion, whereas arginine substitution reduced apoB48 secretion by approximately 45% (Fig. 7*E*). ApoB48 secretion was reduced by greater than 80% by L643E mutation again suggesting that a negatively charged amino acid at position 643 is deleterious to MTP function.

Mutagenesis of L648 to arginine, alanine, glutamic acid, and phenylalanine had no effect on protein expression (Fig. 7*F*, *top*). Substitution of this residue with arginine or glutamic acid significantly reduced PDI binding (Fig. 7*G*). Substitution with phenylalanine had no effect on TG transfer (Fig. 7*H*), but alanine substitution reduced this activity by ∼25% (Fig. 7*H*). Exchange of L648 with glutamic acid or arginine completely abolished TG transfer (Fig. 7*H*). The PL transfer activity trended to increase (∼18%) after phenylalanine substitution and was slightly reduced (∼30%) by alanine substitutions (Fig. 7*I*). Changing L648 to charged residues significantly reduced PL transfer (Fig. 7*I*). Consistent with changes in significant loss of TG and PL transfer activities in L648R and L648E, these mutants were significantly deficient (>70% reductions) in supporting apoB48 secretion (Fig. 7*J*). These studies showed that substitutions at 648 with hydrophobic side chains are well tolerated, but charged residues are not, reinforcing the idea that inner hydrophobic environment of the β1-sandwich is critical for MTP lipid transfer activities and to support apoB48 secretion.

**Figure 8:**
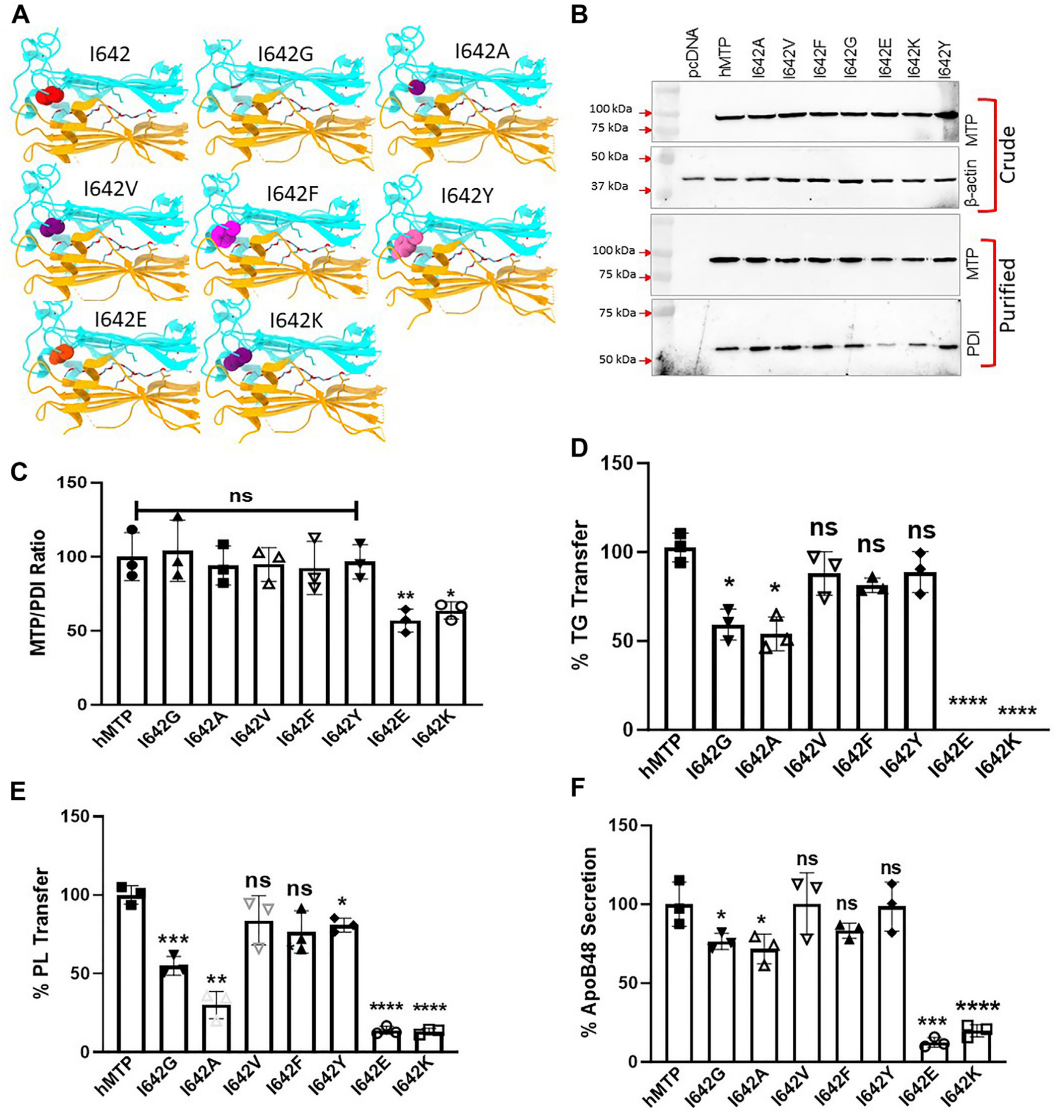
**Effect of different amino acid substitutions at I642 on MTP activity.***A*, the isoleucine residue at 642 position was substituted with different amino acids. Chimera was used to highlight changes in the amino acids at this position. A, alanine; V, valine; F, phenylalanine; G, glycine; E, glutamic acid; K, lysine; and Y, tyrosine. *B*, plasmids expressing WT and different MTP mutants were transfected in triplicates into Cos-7 cells in three different wells. Western blots of MTP and β-actin in crude protein fractions (*top*) and MTP and PDI subunits (*bottom*) in purified MTP variants. *C*, MTP–PDI ratios were calculated from Western blots of purified proteins. *D*, TG transfer activities in purified proteins were measured at 1 h in duplicates. *E*, PL transfer activities in purified proteins were measured at 4 h in duplicate. *F*, ApoB48 concentrations in the media of Cos-7 cells transfected (n = 3) with human apoB48 and different MTP mutants were measured using technical duplicates. All experiments were repeated at least three times, and the multiple comparison was done using one-way ANOVA. The error bars represent SD. ∗*p* < 0.05, ∗∗*p* < 0.01, ∗∗∗*p* < 0.001, and ∗∗∗∗*p* < 0.0001. MTP, microsomal triglyceride transfer protein; PDI, protein disulfide isomerase; PL, phospholipid; TG, triglyceride.

Next, we concentrated on the role of isoleucine 642 in the vicinity of L643 and L648. Mutagenesis of I642 to several amino acids (Fig. 8*A*) had no significant effect on expression (Fig. 8*B*, *top*). Replacing I642 with glycine, alanine, valine, phenylalanine, or tyrosine had no significant effect on PDI binding (Fig. 8*C*); however, substitution of I642 with glutamic acid and lysine residues reduced PDI binding by ∼50% (Fig. 8*C*). We then asked the importance of this hydrophobic residue on lipid transfer activities. Substitution of I642 with amino acids with large hydrophobic side chains valine, phenylalanine, or tyrosine had no effect on TG and PL transfer activities indicating that several amino acids with large hydrophobic side chains are well tolerated in this region (Fig. 8, *D* and *E*). Surprisingly, substitution with shorter hydrophobic residues alanine and glycine reduced TG and PL transfer activities by about 50% (Fig. 8, *D* and *E*). These studies highlighted the importance of large hydrophobic residues in MTP function.

Next, we studied the effect of these mutations on apoB48 secretion in Cos-7 cells (Fig. 8*F*). I642V, I642F, and I642Y mutations had no significant effect on apoB48 secretion. As anticipated, I642E and I642K significantly reduced apoB48 secretion. I642G and I642A decreased apoB48 secretion by about 25%. These studies demonstrated that reducing hydrophobicity at I642 diminishes lipid transfer activities and apoB48 secretion.

### Positive correlation between loss of TG transfer and reductions in apoB48 secretion

**Figure 9:**
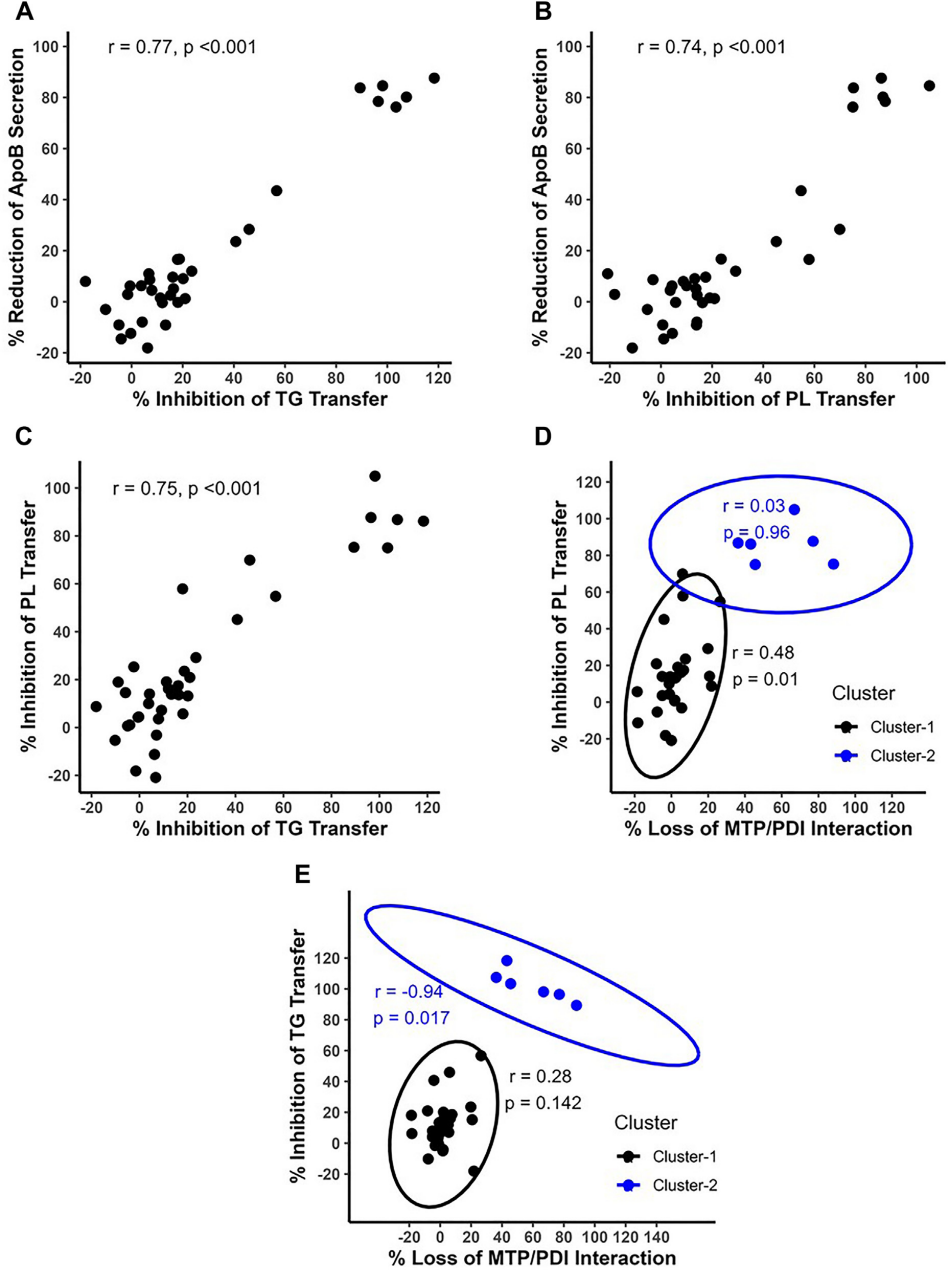
**Correlations amongst different MTP activities.** Data from various mutants described in Figure 3, Figure 4, Figure 5, Figure 6, Figure 7, Figure 8 were used to determine correlations amongst different MTP properties. *A* and *B*, reductions in apoB48 secretion were correlated with reductions in (*A*) TG and (*B*) PL transfer activities. *C*, relationship between loss of TG and PL transfer activities. *D* and *E*, the % loss of MTP–PDI interactions were correlated with reductions in PL (*D*) and TG (*E*) transfer activities using K-means clustering and Pearson correlation. MTP, microsomal triglyceride transfer protein; PDI, protein disulfide isomerase; PL, phospholipid; TG, triglyceride.

In this study, different missense mutations inhibited TG and PL transfer activities of MTP to different extents. Therefore, we correlated these inhibitions with reductions in apoB48 secretion. There was a significant positive correlation between loss of TG and PL transfer activities and reductions in apoB secretion (Fig. 9, *A* and *B*). Furthermore, when the results for the mutations were analyzed for the loss of both TG and PL transfer activities, there was a strong correlation between changes in TG and PL transfer activities; that is, when one activity is inhibited, the other activity is also diminished (Fig. 9*C*). This reinforces the concept of a common binding site for both these lipids.

Next, we attempted to correlate changes in PL (Fig. 9*D*) and TG (Fig. 9*E*) transfer activities of MTP with loss of PDI binding. Preliminary correlation plots between loss of MTP–PDI interactions and loss of lipid transfer activities suggested the presence of distinct clusters, prompting further analysis (Fig. 9, *D* and *E*). Employing the K-means clustering technique (42), we identified two distinct clusters in the loss of both PL and TG activities that were differentially related with the loss of MTP–PDI interactions (Fig. 9, *D* and *E*). The loss of transfer activities in cluster 1 was independent of loss of MTP–PDI interactions, whereas in cluster 2, the loss of transfer activities was associated with loss of MTP–PDI interactions. We interpret these data to suggest that loss of lipid transfer activities could occur by two independent mechanisms. In cluster 1, different mutants lose lipid transfer activities because the mutated amino acid residues are important for lipid transfer. In cluster 2, loss of lipid transfer activities may be secondary to loss of MTP–PDI interactions.

### Discrepancies between predictive algorithms and experimental determination of MTP functions

We compared our results with several algorithms (SIFT, Panther, and Polymorphism Phenotyping [PolyPhen]) that predict effect of substitutions on protein activity (Table S1). Most of the predicted “benign” or “tolerated” mutations had no effect on various MTP functions studied here. However, several mutations predicted to be “probably damaging” and “not tolerated” had no effect on experimentally determined MTP functions. On the other hand, all the mutations that showed defects in MTP functional studies were predicted to be “probably/possibly damaging” and “not tolerated” by different algorithms. These studies suggested that these algorithms are more prone to predict disruption in protein function.

## Discussion

The aim of this study was to garner information about the lipid-binding sites for TG and PL in MTP. Our strategy was to substitute one amino acid at a time to avoid protein misfolding. We performed control experiments, such as mobility on polyacrylamide gels and PDI binding, to learn about major structural changes. Most of the mutations had no effect on the mobility of MTP protein in polyacrylamide gels indicating no gross structural changes affecting mobility. Similarly, many of the mutants had no effect on PDI binding. However, we did identify several mutations that affected PDI binding but had no effect on gel mobility. Nevertheless, we cannot rule out the possibility of subtle local changes and whether these changes were responsible for lower lipid transfer activities in some mutants, such as L643A, L643G, L648A, or L648G.

We had hypothesized, because of differences in size and charge between them, that there may be two distinct sites, with TG being bound by β1-sandwich and PL being bound by β2-sandwich. Furthermore, we had hypothesized that positively charged amino acids would be important for PL transfer.

Our biochemical studies revealed that this is not the case. Instead, we found that positively charged residues in the β1- and β2-sandwiches are not critical for PL transfer and that the transfer activities for both lipid types are similarly affected by mutagenesis of hydrophobic amino acids in the β1-sandwich. Thus, our data indicate that MTP contains one hydrophobic β1-sandwich that is critical for both TG and PL lipid transfer activities, and targeting of this domain may inhibit all transfer activities.

The substitution of charged residues with neutral residues in β1- and β2-sandwiches neither enhanced nor diminished lipid transfer activities. There could be several explanations for these unanticipated results that positively charged amino acids do not play a role in PL transfer. Our prediction of the PL transfer domain might be wrong and that other charged residues at different locations might be involved. Negative charges in lipids may not be important in PL transfer; instead, MTP may only recognize hydrophobic patches as has been predicted before (8). More experiments are needed to identify determinants of PL transfer in MTP. Identification of such a domain may be facilitated by crystallization of MTP with PLs.

Identification of a common hydrophobic domain for the transfer of TG and PL was unanticipated because of significant differences in these lipids with respect to hydrophobic indices and charges. It is likely that this domain binds hydrophobic fatty acids present in TG and PL. Since this domain cannot tolerate charges in amino acid side chains, it is unlikely that this region could accommodate charged groups in PL. Therefore, more studies are needed to learn how MTP accommodates and transfers different PL.

Mutagenesis of L642, L643, and L648 revealed that these residues can be safely replaced with amino acids with large hydrophobic side chains, such as phenylalanine, without affecting MTP function. Surprisingly, substitution of leucine with glycine and alanine residues had partial inhibitory effect on lipid transfer activities. These studies provide novel information that bulky hydrophobic side chains are critical for lipid transfer. It is likely that a large hydrophobic domain is critical for lipid transfer. These studies also provided some information about the importance of different lipid transfer activities in apoB48 secretion. There is a positive correlation between inhibition of TG and PL transfer with reductions in apoB48 secretion highlighting the importance of these lipid transfer activities in apoB secretion (Fig. 9).

Our studies show that several mutations predicted to be damaging and “not tolerated” by online algorithms had no significant effects on MTP function when experimentally determined. The reasons for these discrepancies are not clear, but it is likely that MTP function may be more resistant to structural changes than predicted by these algorithms. It is possible that mutations that may otherwise be predicted to be structurally damaging can perhaps be accommodated in the protein to preserve function. In other words, MTP may be resistant to subtle structural perturbations and is a more flexible protein that can accommodate these insults than predicted by algorithms. This is not surprising based on its vital and nonredundant function in supporting the transfer of energy to different tissues.

A caveat of this study is a limited focus on two structural domains to identify TG and PL transfer domains. Data presented here suggest that there may not be two distinct transfer domains for these lipids. Instead, multiple regions and domains may participate in the transfer of different lipids. As explained earlier, our results are in discordance with our studies with G865V (22) and other biochemical studies (23). These studies point to the possible existence of a common binding site that perhaps recognizes hydrophobic fatty acid chains present in TG and PL. There might be additional determinants responsible for differential binding affinities for TG and PL. It is possible that positive charges do not play a role in PL recognition, but interactions of phosphate groups in PL with -OH groups of serine and threonine residues might be important. Therefore, more studies are needed to recognize all common and specific determinants that recognize and discriminate different lipids.

More studies are needed to identify critical residues in PL transfer. Another caveat of this study is that the knowledge obtained here is solely based on cell culture and *in vitro* studies. Further studies in animal models may provide more comprehensive information about the role of different domains and amino acids in MTP function.

In summary, crystal structure–based interrogation of different domains *via* site-directed mutagenesis revealed that positively charged amino acids in β2-sandwich domain are not critical for PL transfer. These studies, however, identified that amino acids with large hydrophobic side chains in the A- and C-sheets of β1-sandwich are important for both TG and PL transfer activity. Thus, MTP contains a common hydrophobic domain that can accommodate different lipids. It is likely that this region is essential for MTP function and should be avoided in drug development.

## Experimental procedures

### Materials

Monkey kidney Cos-7 cells and the plasmids pcDNA3 and pcDNA3-*hMTP-FLAG* were as described previously (8, 14, 15, 20, 21, 33, 38). Oligonucleotides were obtained from Integrated DNA Technologies. LB agar (catalog no.: 110283) and LB broth (catalog no.: 110285) for microbiology work were purchased from Millipore. Anti-FLAG M2 affinity agarose gel (catalog no.: A2220-5Ml), monoclonal anti-FLAG M2 antibody (catalog no.: F3165), carbenicillin disodium salt (catalog no.: C3416-5G), and other chemicals were purchased from MilliporeSigma. FLAG peptide was custom synthesized (GenScript). Round bottom black 96-well assay plates were obtained from Costar (catalog no.: 3792; Kennebunk). Human apoB monoclonal antibody, 1D1, was amplified by MyBiosource (catalog no.: MBS465020) for ELISA. Swine anti goat-IgG antibody from Southern Biotech (catalog no.: 6300-04), goat polyclonal anti-hApoB antibody from Academy Bio-Medical Company, Inc (catalog no.: 20S-G2-1.5Ml), and 96-well ELISA assay plates from Corning (catalog no.: 3366) were purchased.

### Site-directed mutagenesis

We used site-directed mutagenesis to generate point mutations/substitutions in human *MTTP* complementary DNA cloned in pcDNA3 plasmid as described earlier (8, 15, 38). In brief, the forward and reverse primers (Table S2) for each mutation were designed using NEBase changer online tool from New England Biolab (NEB) website as per their protocol. The Q5 site-directed mutagenesis kit (catalog no.: E0554; NEB) was used for PCR amplification of pcDNA3-*MTTP-FLAG*. The PCR amplification was confirmed by resolving the PCR product on 0.8% agarose gel. Then, 1 μl (50–100 ng) of the PCR product was subjected to KLD enzyme reaction as suggested in the kit, and the product was transformed into *Escherichia coli*–competent cells supplied in the kit. The transformants were selected on LB-agar plates containing 100 μg/ml of carbenicillin antibiotic. Plasmid was isolated from antibiotic-resistant *E. coli* colonies, and DNA sequencing (Genewiz) was performed to confirm the presence of desired mutation. Colonies with the desired mutations were grown in 200 ml LB broth, and the plasmid was isolated using ZymoPURII plasmid maxi prep kit (catalog no.: D4204; Zymo Research).

### Transfection of Cos-7 cells with expression plasmids using chemical reagent

To express various MTP mutants, the pcDNA3 control and pcDNA3 carrying FLAG-tagged MTP mutant complementary DNAs were transfected into Cos-7 cells using EndoFectin Max transfection reagent (Genecopoeia; catalog no.: EF013) as described earlier (14, 15, 33, 34). We did not express PDI. Expressed MTP subunit interacts with endogenous PDI to form an active complex. In brief, Cos-7 cells were seeded in T75 flasks and allowed to grow till 90% confluency. Next, cells were trypsinized and washed with Opti-MEM media (Gibco; catalog no.: 31985070), and 1 × 10^6^ cells were resuspended in 5 ml of Opti-MEM media. The plasmid (9 μg) was mixed with EndoFectin (2 μl of EndoFectin/1 μg of plasmid) in 0.5 ml Opti-MEM media. The mixture was incubated at room temperature for 10 min and added to 100 mm cell culture dishes followed by cells. After 12 h of incubation in CO_2_ incubator at 37 °C, the Opti-MEM media were removed, and Dulbecco's modified Eagle's medium containing serum was added.

### Preparation of cell homogenates and purification of MTP protein

The transfected Cos-7 cells were grown for 48 h and then harvested by scrapping from petri dishes in 1 ml of buffer K (10 mM Tris–Cl, 150 mM NaCl, 1 mM MgCl_2_, and 1 mM EGTA, pH 7.4) (15, 33). The harvested cells were subjected to sonication on ice with 35% amplitude, 2 s pulse on and 1 s off, for 90 s (550 Sonic Dismembrator; Fisher Scientific). The cell homogenates were centrifuged at 12,000 rpm or 13,500*g* in a centrifuge (catalog no.: 5424R; Eppendorf) for 10 min at 4 °C to remove unbroken cells and cell debris. The clear supernatants were collected in fresh sterile tube and stored in refrigerator at 4 °C for 1 to 2 days or used immediately for further experiments. The protein concentration was quantified using a Pierce BCA protein assay kit (catalog no.: 23225; Thermo Scientific) and bovine serum albumin as standard. The MTP purification was done as described earlier (15, 33). In brief, about 500 μg of cell protein lysate was rotated with 50 μl of anti-FLAG M2 affinity gel (Sigma; catalog no.: A2220-5Ml) overnight at 4 °C, washed thrice with ice-cold buffer K, and MTP was eluted in 500 μl buffer K containing 100 μM of FLAG peptide. For negative control, protein was extracted and purified from Cos-7 cells transfected with only pcDNA3 control plasmid.

### MTP activity assay

The TG and PL transfer activities of MTP mutants were measured in both cell homogenates and purified proteins. We used NBD-TG (1,3-Diolein, 2-NBD-X ester; Setareh Biotech, catalog no.: 6285) and NBD-phosphatidylethanolamine (Avanti Polar Lipids; catalog no.: 810145C) as representative of TG and PL transfer, respectively (33, 36). The high concentration of NBD-TG or NBD-PL in respective donor vesicles results in self-quenching. When these lipids are being transferred by MTP, they fluoresce, and increases in fluorescence are measured as MTP activity (33, 36). The PL transfer activity was measured as described earlier (33) in triplicate with a mixture of 5 μl of acceptor vesicles and 5 μl of donor vesicles containing NBD-PL in a total volume of 100 μl. First, the donor and acceptor vesicles were added to the wells of a 96-well black round bottom microtiter plate (Costar; catalog no.: 3792), and then 90 μl of sample containing either crude (50 μg) or purified (90 μl) protein was added. For blank controls, only buffer K was added to the vesicles. For total fluorescence measurements, isopropanol (90 μl) was added to a mixture of donor and acceptor vesicles (10 μl). For TG transfer activity, 5 μl mixture of donor and acceptor vesicles containing NBD-TG (2.5 μl AV + 2.5 μl DV) was added to microtiter plates. Next, 95 μl of buffer K or isopropanol was added and immediately started measuring NBD fluorescence. The NBD fluorescence of both the TG and PL transfer assays was measured at λ_excitation_ 463 nm and λ_emission_ 536 nm in an Enspire plate reader (PerkinElmer) (33). The PL transfer activity was monitored for 240 min, and TG transfer activity was monitored for 60 min. Both assays were performed at room temperature. The samples used for activity measurements were also subjected to Western blot analyses to quantify amounts of WT and mutant MTP protein levels. The lipid transfer activities were normalized to adjust for small differences when present in protein levels.

### Immune detection of MTP and PDI

The protein homogenates from MTP-expressing Cos-7 cells and purified MTP proteins were used for immune detection of MTP and PDI. About 25 μg of crude protein and 30 μl of purified MTP protein were resolved on 8% SDS-PAGE and blotted on to nitrocellulose membrane using *Trans*-Blot Turbo Transfer System (catalog no.: 1704150; Bio-Rad). The MTP was detected using mouse monoclonal anti-FLAG antibody (catalog no.: F3165; Sigma). The interactions of different MTP mutants with PDI subunit were studied in purified proteins by Western blotting. The purified proteins were separated on polyacrylamide gels, transferred to nitrocellulose membranes, and first probed with anti-FLAG antibodies. After stripping, these blots were processed to detect PDI monomer using mouse monoclonal anti-PDI antibodies (Invitrogen; catalog no.: RL77). Beta-actin, a loading control, was detected using rabbit polyclonal anti β-actin antibodies (Cell Signaling; catalog no.: 4967S).

### ApoB measurements in the media by ELISA

The ability of different MTP mutants to drive the secretion of apoB48 from Cos-7 cells was assessed by ELISA as before (15, 34, 35). The Cos-7 cells (5 × 10^7^) were forward transfected in 150 mm plates with 20 μg plasmid (pCMV-hApo48) for the expression of human apoB48. After 24 h, the trypsinized cells (400,000 cells/well, 6-well plate) were reverse transfected with 3 μg of pcDNA3 plasmid carrying different hMTP mutants and allowed to grow for 48 h. The media were changed 16 h before the collection of samples for ELISA. The media were centrifuged (10,000*g*, 5 min, 4 °C) to remove dead cells and any particulate debris. The supernatants were diluted appropriately and stored at −80 °C freezer until use. The ELISA plate was coated with 100 μl of capture antibody (mouse monoclonal anti-ApoB antibody, 1D1, 1 mg/ml, 1:1000 dilution in PBS) and incubated overnight at 4 °C. Next morning, the plate was washed thrice with 350 μl of PBS containing 0.02% Tween-20 buffer and blocked with 100 μl of 3% bovine serum albumin solution in PBS for 1 h at room temperature. Then, plate was washed again as described previously, 100 μl of diluted and/or undiluted samples were loaded along with 0 to 100 ng of LDL standard in different wells, and incubated at 37 °C for 2 h. The plate was washed thrice with PBS with Tween-20 buffer, 100 μl of detection antibody (goat polyclonal anti-ApoB antibody, 1:1000 dilution) was added to each well, and incubated for another 1 h at 37 °C. Again, the plate was washed thrice, and 100 μl of secondary antibody (swine polyclonal antigoat IgG antibody, 1:2000 dilutution) was added to each well and further incubated for 1 h at 37 °C. Finally, the plate was washed and developed by adding 100 μl 1 mg/ml (w/v) of p-nitrophenyl phosphate substrate dissolved in substrate buffer (100 mM glycine, 1 mM MgCl_2_.6H_2_O, and 1 mM ZnCl_2_, pH 10.4). The plate was incubated in dark for 5 to 10 min or till the yellow color developed. The yellow color was measured at 405 nm. The concentration of apoB in samples was calculated using the LDL standard.

### Bioinformatics and statistical analysis

The MTP structure was viewed and analyzed using UCSF Chimera (Resource for Biocomputing, Visualization, and Informatics at the University of California, San Francisco) (43) and Phyre2 (44) software. The plasmid DNA sequence and chromatogram were visualized by SnapGene. The predicted effects of mutations on MTP function were assessed using online bioinformatics tools; SIFT (45), Panther (46), and PolyPhen (47). The SNP sequence was searched and downloaded from the National Center for Biotechnology Information SNP database. The percentage transfer (%T) of TG and PL was calculated as previously described (33, 36, 37) with the formula [%T = (Fs-Fb)/(Ft-Fb) × 100], where Fs is the fluorescence of the test sample, Fb is the fluorescence of the blank, and Ft is total fluorescence. The density of the protein bands on Western blots was quantified using ImageJ software (National Institutes of Health). All graphing and statistical analyses were performed in GraphPad Prism software, version 8.4.3 (GraphPad Software, Inc). One-way ANOVA with Dunnett’s multiple comparisons was used to compare different mutations using hMTP as the control. Results were considered statistically significant if *p* < 0.05. All correlation studies used SAS 9.4 (SAS institute Inc) and R 4.3.2 (R Foundation for Statistical Computing). Using histogram and scatter plots, our preliminary analyses suggested deviation from normality in the data distribution with respect to loss of lipid transfer activities and MTP–PDI interactions. In addition, the plots suggested the presence of distinct clusters. Employing the K-means clustering technique, we identified two clusters. The Spearman correlation analysis was performed within each cluster when assessing relationship between loss of MTP–PDI interactions and loss of lipid transfer activities.

## Data availability

All the data are in the article.

## Supporting information

This article contains supporting information..

## Conflict of interest

The authors declare that they have no conflicts of interest with the contents of this article.
